# Evaluation of single nucleotide polymorphisms in 6 candidate genes and carotid intima-media thickness in community-dwelling residents

**DOI:** 10.1371/journal.pone.0230715

**Published:** 2020-03-26

**Authors:** Fang-Yang Wu, Chia-Ing Li, Li-Na Liao, Chiu-Shong Liu, Wen-Yuan Lin, Chih-Hsueh Lin, Chuan-Wei Yang, Tsai-Chung Li, Cheng-Chieh Lin

**Affiliations:** 1 Department of Public Health, College of Public Health, China Medical University, Taichung, Taiwan; 2 School of Medicine, College of Medicine, China Medical University, Taichung, Taiwan; 3 Department of Medical Research, China Medical University Hospital, Taichung, Taiwan; 4 Department of Family Medicine, China Medical University Hospital, Taichung, Taiwan; 5 Department of Healthcare Administration, College of Medical and Health Sciences, Asia University, Taichung, Taiwan; University of North Carolina at Chapel Hill, UNITED STATES

## Abstract

Evidence suggests the existence of association between a large panel of modifiable biomarkers representing inflammation, coagulation, paraoxonase, and endothelial activation pathways and carotid atherosclerosis. Thus, this study investigated whether *CRP*, *FGA*, *FGB*, *FGG*, *PON1*, and *EDNRA* gene variants affected plasma hs-CRP, fibrinogen levels, and thickness of carotid intima media thickness (IMT). Nineteen single-nucleotide polymorphisms of *CRP*, *FGA*, *FGB*, *FGG*, *PON1*, and *EDNRA* genes were examined in 480 participants from 160 families. Carotid IMT was measured by ultrasound. Generalized linear models with generalized estimating equation were utilized to consider the dependence of subjects within families. In the recessive model, homozygotes for the minor alleles of rs1800789, rs1800790 and rs4220 SNPs in *FGB* gene indicated a reduced risk of IMT (Exp. β = 0.89, 0.89, 0.88), which remained significant after adjustment for confounding factors. Significant interaction effects between *CRP* SNP rs1130864 and rs3093059 and gender for IMT were observed with a significant association in men only. Men carrying minor-minor genotype of *CRP* SNP rs1130864 and rs3093059 had 0.70- and 0.78-fold lower IMT than men carrying minor-major/major-major genotype. We also observed that the interaction of *CRP* SNP rs1130864 and rs3093059 with obesity on IMT, hs-CRP and fibrinogen levels. These results support the hypothesis that inflammatory genes are involved in atherosclerosis, most likely via complex gene-gender and gene-obesity interactions.

## Introduction

Carotid intima-media thickness (IMT) is one of the best established and most commonly used early surrogate markers of atherosclerosis [1]. Carotid IMT has recently emerged as an important predictor for cardiovascular events in the general population [2] and in asymptomatic subjects with multiple cardiovascular disease (CVD) risk factors [3]. The Atherosclerosis Risk in Communities (ARIC) study show an increase of 0.19 mm in the carotid IMT, an increase of 36%-69% in the risk of coronary disease, and an increase in the risk of stroke by 3.6–8.5 times [4, 5].

Atherosclerosis is one kind of chronic inflammatory disease. Inflammation and endothelial injury play pivotal roles in the pathogenesis of atherosclerosis [6, 7]. Endothelial injury affects the balance between vasodilation and vasoconstriction [8]. In order to repair the endothelial tissue, fibrinogen was cleaved by thrombin to form fibrin. Low-density lipoprotein (LDL) accumulates at the site of endothelial injury and may become oxidized [9], and further induce inflammation, although paraoxonase 1 (PON 1) stimulate the antioxidant activity of high-density lipoprotein (HDL) to fight the oxidized- LDL [10]. Accumulation of macrophages at the site of endothelial injury regulate the endothelin A receptors shift to endothelin B receptors and enhance endothelin system, and further express C-reactive protein (CRP) mRNA contribute to the progression of atherosclerosis [11, 12]. These six genes are involved in the development of atherosclerosis, including *CRP*, endothelin receptor type A (*EDNRA*), *PON 1*, fibrinogen alpha chain (*FGA*), fibrinogen beta chain (*FGB*), and fibrinogen gamma chain (*FGG*).

Many prior studies have demonstrated that the increase in IMT is associated with most of the known CVD and atherosclerotic risk factors, such as CRP, fibrinogen, PON 1, and EDNRA levels or gene variants [13–19], although those associations have not been confirmed in some studies [20, 21]. Some studies indicated a modest graded relation between CRP and carotid plaque and carotid IMT in different populations [13, 19, 22]. The studies of CRP and IMT confirmed that increased CRP is a risk factor for ischemic stroke, and atherosclerosis is measured by carotid IMT degree, i.e. the increase in IMT also increases the risk of atherosclerosis [13, 19].

Fibrinogen plays an important role in blood coagulation and is also a key regulator of inflammation processes [23]. Elevated fibrinogen level is a well-characterized risk factor for CVD [24]. Prior studies indicated that increased fibrinogen was correlated with carotid IMT in adults without clinically overt atherosclerotic disease [17, 18]. Studies on genetic determinants of fibrinogen typically focused on the *FGA*, *FGB*, and *FGG* genes. However, polymorphisms of these genes denoted only a small part of the estimated heritable effects on fibrinogen [23, 25, 26]. Reports show inconsistent evidence of the association between *CRP* and fibrinogen gene variants and carotid IMT [27, 28].

PON1 degrades oxidized lipids. Previous studies revealed the association between the low plasma PON1 activity and the increase CVD risk regardless of age and ethnicity [29, 30]. Thus, PON1 activity is genetically adjusted and differs widely among populations. Cece et al. study showed that decreased PON1 activity and increased IMT suggest significantly relationship in subjects with atherosclerosis and ankylosing spondylitis [14]. Similarly, a negative relationship was observed between PON1 activity and IMT in individuals with coronary heart disease [31]. As for EDNRA protein, its existence is found in the membrane of vascular smooth muscle cells [32]. The proliferation and migration of smooth muscle cells from the media into the intima mediated by EDNRA is significant with atherosclerosis development and progression [33, 34]. Little is known regarding the relationship between *EDNRA* gene and carotid IMT [15, 16]. Li’s study indicated the interaction of smoking with *EDNRA* gene variation on left carotid IMT in Africa Americans [15], whereas Lopez-Mejias’s study revealed no significant differences in carotid IMT according to *EDNRA* gene polymorphisms in patients with rheumatoid arthritis [16]. In our previous analyses of this study population, genetic polymorphisms of *EDNRA* revealed a significant interaction with regular exercise or obesity on IMT [35].

Carotid IMT is related to a family history of pre-clinical carotid artery cardiovascular disease [36]. The measurements of carotid IMT is a good quantitative phenotype for the assessment of its genetic determinants. Prior studies estimated the heritability of carotid IMT, thereby demonstrating great variation depending on the population. Most family studies revealed the heritability between 0.30 and 0.60, thereby indicating that genetic factors play a key role in carotid IMT [37–40]. This study was designed to investigate the association between a large panel of modifiable biomarkers representing inflammation, coagulation, paraoxonase, and endothelial activation pathways and carotid IMT. In any complex polygenic event, the overall influence of each gene is probably small and may be masked by the effect of gene-environment interactions. Therefore, we hypothesized that the tag single nucleotide polymorphisms (SNPs) of *CRP*, *FGA*, *FGB*, *FGG*, *PON1*, and *EDNRA* genes are related to higher high-sensitivity CRP (hs-CRP) and fibrinogen levels and to increased thickness of IMT. We also hypothesized that their effects would vary according to environmental factors, such as gender, obesity, and regular exercise.

## Methods

### Study subjects

This study was conducted after obtaining approval from the Institutional Review Board of China Medical University Hospital and all methods were performed in accordance with the relevant guidelines and regulations. Written informed consent was obtained from each participant. The study conforms to the recognized standards (Declaration of Helsinki). A community-based family study was conducted to explore the relationship between genetic variants and subclinical atherosclerosis [35]. All proband cases and proband controls were identified from our cohort of Taichung Community Health Study (TCHS). The TCHS was conducted in 2004 and it consisted of 2,359 participants who were randomly selected from among Taichung’s general population aged ≥40 years old. A total of 1,666 subjects were followed up, and the over-all follow-up rate was 73.3%. Our analyses are based on carotid IMT measurements collected at the second wave of the follow-up evaluations, which were conducted between 2006 and 2009. We invited family members of study subjects from TCHS individually by letter and by phone to participate in our genetic study. This family study recruited 484 TCHS participants and 1080 family members to estimate the familiar aggregation and heritability of specific cardiovascular risk phenotypes in our prior study. In this genetic study, we included 80 proband cases and 80 proband controls with their spouses and one first-degree blood relative aged 20 years old and older for this genetic study (a total of 480 people). Proband cases were those who had their maximum IMT (IMT_max_) in the area of the common carotid arteries (CCAs), carotid bulb, and internal carotid artery (ICA) ≥ 1.5 mm, and IMT_max_< 1.5 mm as proband controls, whereas proband control had their IMT ranges from 0.25 to 1.5 mm [41]. The inclusion criterion for this genetic study was that study subjects must have more than one first-degree relatives.

### Measurements

A standardized questionnaire was designed to collect data on sociodemographic characteristics, including gender, age, education, smoking, drinking, regular exercise, betel nut chewing, family and personal history of cardiovascular-related diseases, and medication history. The questionnaire used in this study was shown in Supplemental Information. Hypertension, diabetes, and hyperlipidemia were defined as the use of medications or self-reported. Anthropometric measurements were obtained from physical examination. Weight and height were measured on an autoanthropometer (super-view, HW-666), with no shoe and light clothing. Body mass index (BMI) was defined as weight (kg) ÷ (height)^2^ (m) ^2^. Obesity was defined as BMI > = 27 kg/m^2^. Study subjects who had regular exercise were those who participated in regular leisure-time activities for at least 30 min per week for at least 6 months when they were interviewed. Venous blood samples were obtained after 12-hour overnight fasting. Biochemical blood tests such as total cholesterol (TC), high density lipoprotein (HDL)-cholesterol, low density lipoprotein (LDL)-cholesterol, triglyceride (TG), urine albumin, creatinine, uric acid, blood urea nitrogen (BUN), fasting plasma glucose (FPG), and insulin levels were measured by using a biochemical autoanalyzer (Beckman Coluter, Lx-20, USA) at the Clinical Laboratory Department of China Medical University Hospital. Hs-CRP and fibrinogen levels was measured using nephelometry, a latex particle-enhanced immunoassay (TBA-200FR, Tokyo, Japan). The inter-assay and intra-assay coefficient of variation (CV) for hs-CRP are <2.0% and 1.9%, respectively. The lower detection limit of the assay is 0.1 mg/L. The inter-assay and intra-assay CVs for fibrinogen are 3.6% and 6.1%, respectively.

All study subjects were measured by the same machine (GE L7000, GE, Milwaukee, Wis., USA). After having a rest for at least 10 min in the supine position with the neck in slight hyperextension, all study subjects underwent carotid ultrasound examination using 7.5-MHz probe to scan the near and far wall of arterial segments bilaterally in order to get the longitudinal (anterior oblique, lateral, and posterior oblique) and transverse views. Each ultrasound image was recorded on a computer with an online digital filing system, and the intima-media complex thickness and atherosclerotic plaques were measured. The 12 arterial segments involves 1) 1 to 2 cm proximal to the tip of the flow divider into the common carotid arteries (CCAs); 2) the carotid bifurcations beginning at the tip of the flow divider and extending 1 cm proximal to the flow divider tip; and 3) the proximal (first 1 cm) of the internal carotid arteries (ICAs). Maximal IMT of each segment at the CCA was determined from a minimum of 3 frames as the maximum IMT (M-IMT) of each segment by averaging these frames. The total carotid IMT was calculated as a composite measure (mean of the 8 carotid sites) that combine the near and the far wall of the CCA IMT, the bifurcation IMT, and the ICA IMT of both sides of the neck. Total IMT were expressed in a mean of the maximums of the 8 carotid sites.

Genomic deoxyribonucleic acid (DNA) was extracted from peripheral blood leucocytes by using QIAamp Blood Kit (Qiagen, Chatsworth, CA, USA). DNA concentration was measured by using a NanoDrop ND-2000c spectrophotometer (NanoDrop Technologies, Wilmington, DE, USA). The purity of genomic DNA was verified by evaluation of OD260/OD280 ratio. The criteria used for SNPs selection were as follows: 1) A minimum of 60 bases is required for upstream or downstream flanking region of each SNP with another SNP; 2) Minor allele frequency of each SNP must be 5% or greater in the HapMap dataset (Han Chinese in Beijing (CHB) population); and 3) The final score of each SNP must be 0.6 or greater by using the Illumina Assay Design Tool. We selected the *CRP* (SNP rs12093699, rs876537, rs1205, rs1130864, and rs3093059), *FGA* (SNP rs2070016 and rs2070011), *FGB* (SNP rs1800789, rs1800790, rs1800788, and rs4220), *FGG* (SNP rs7659024, rs1049636, and rs7681423), *PON1* (SNP rs854555 and rs662), and *EDNRA* (SNP rs1395821, rs1878406 and rs5333) genes based on the findings on the CHB population in the HapMap dataset (CHB population). These polymorphisms were detected by using Illumina GoldenGate Genotyping Assay (Illumina, San Diego, CA, USA). Each of the genomic DNA samples was amplified and the polymerase chain reaction products were hybridized to VeraCode microbeads and detected on a VeraCode BeadXpress Reader. SNP genotypes were typed by GenomeStudio Data Analysis software. The quality controls of GoldenGate Genotyping Assay were verified in each steps, such as allele-specific extension, first hybridization, PCR uniformity, extension gap, and second hybridization.

### Statistical analysis

The values of mean and standard deviation (SD) for continuous variables and number and proportion for categorical variables were presented stratified by proband type (case and control) and type of family member (proband, spouse, and offspring). Nineteen SNPs were assessed for deviation from Hardy-Weinberg equilibrium (HWE) using PLINK software. Covariates for IMT were age, gender, BMI, waist-hip ratio (WHR), hs-CRP, fibrinogen, lifestyle behaviors, biochemical markers (including FPG, TC, HDL, and TG), and disease history (hypertension, diabetes, hyperlipidemia, cardiovascular disease, and cancer). The average values of IMT, hs-CRP, and fibrinogen were compared among genotypes by using generalized linear model(s) (GLM) with generalized estimating equation(s) (GEE) to account for the dependence of subjects within families. Because distributions of Max IMT, hs-CRP, ACR (albumin to creatinine ratio), and TG skewed to the right, natural log-transformation for these markers was used to normalize data and geometric means of these markers values and SDs were reported. Estimated coefficient of regression and standard error (SE) were calculated. Genotypic models were used for the single-SNP association analysis. Because most SNPs exerted recessive effects, recessive model was adopted for multivariate models examining gene-environment interactions. Gene-environment interactions containing gene-gender, gene-obesity, and gene-regular exercise interactions on carotid IMT, hs-CRP, and fibrinogen were evaluated by GLM with GEE adjusted for confounders. Then, interaction plots were presented for those analyses with significant interactions. A significant gene-environment interaction was interpreted as “a different effect of a genotype on disease risk in persons with different environmental exposures” [42]. The p-values corrected for false discovery rate method were reported to consider multiple testing problem. All analyses used Statistical Analysis System software (v9.4, SAS Institute Inc., Cary, NC, USA) and PLINK (v1.07) (http://pngu.mgh.harvard.edu/purcell/plink) [43].

## Results

### Basic characteristics of probands, spouses, and offspring

The characteristics of study subjects, consisting of 80 proband cases and 80 proband controls along with their spouses and offsprings, are shown in Table 1. Mean ages were 63.0 and 59.8 years old for proband cases and controls, 61.2 and 59.5 years old for their spouses, and 33.9 and 30.9 years old for their offspring. Males accounted for 70% and 50% of proband cases and controls, 30% and 50% of their spouses, and 60% and 53.8% of their offspring. The number of individuals with max IMT ≥1.5 mm for proband cases and controls were 80 and 0, whereas those for their spouses were 30 and 28, and those for their offspring were 5 and 2, respectively. Mean values of BMI, IMT, ACR, and proportions of smoking, hypertension, heart disease, and cerebral vascular accident (CVA) were higher in proband cases than in the other five groups.

**Table 1:** Characteristics of study subjects.

	Proband case family (n = 80)			Proband control family (n = 80)		
Characteristic	Case (IMT ≥1.5 mm)	Spouse	Offspring	Control (IMT <1.5 mm)	Spouse	Offspring
Age (years)	63.0 ± 6.0	61.2 ± 7.1	33.9 ± 6.8	59.8 ± 6.3	59.5 ± 7.1	30.9 ± 6.7
Male	56 (70.0)	24 (30.0)	48 (60.0)	40 (50.0)	40 (50.0)	43 (53.8)
BMI (kg/m^2^)	24.9 ± 3.3	24.2 ± 3.2	23.7 ± 4.1	24.3 ± 2.6	23.8 ± 3.0	22.6 ± 3.5
Obesity (BMI ≥27 kg/m^2^)	16 (20.0)	16 (20.0)	15 (18.8)	12 (15.0)	10 (12.5)	8 (10.0)
WHR	0.9 ± 0.1	0.9 ± 0.1	0.8 ± 0.1	0.9 ± 0.1	0.9 ± 0.1	0.8 ± 0.1
Max IMT (mm)^#^	2.1 ± 1.3	1.4 ± 1.5	0.9 ± 1.4	1.1 ± 1.2	1.3 ± 1.5	0.7 ± 1.3
Max IMT ≥1.5 mm	80 (100.0)	30 (37.5)	5 (6.3)	0 (0.0)	28 (35.0)	2 (2.5)
Hs-CRP (mg/dL)^#^	0.1 ± 3.0	0.1 ± 2.7	0.1 ± 2.9	0.1 ± 2.6	0.1 ± 2.6	0.1 ± 2.7
Fibrinogen (mg/dL)	363.4 ± 71.4	363.8 ± 79.8	351.7 ± 71.3	370.0 ± 72.0	353.6 ± 54.8	336.9 ± 63.2
**Behaviors**						
Physical activity	59 (73.8)	61 (76.3)	36 (45.0)	58 (72.5)	59 (73.8)	33 (41.3)
Smoking (Yes/ever)	25 (31.3)	12 (15.0)	16 (20.0)	4 (5.0)	17 (21.3)	10 (12.5)
Alcohol drinking (Yes/ever)	20 (25.0)	13 (16.3)	12 (15.0)	17 (21.3)	17 (21.3)	14 (17.5)
Betel nut chewing (Yes/ever)	5 (6.3)	2 (2.5)	1 (1.3)	1 (1.3)	2 (2.5)	2 (2.5)
**Biochemical marker**						
FPG (mg/dL)	108.7 ± 32.2	104.1 ± 19.0	94.5 ± 9.0	105.8 ± 25.5	105.5 ± 28.0	96.1 ± 21.5
Insulin (mg)	6.8 ± 3.6	6.1 ± 3.9	7.1 ± 4.3	6.5 ± 5.4	6.2 ± 3.7	6.6 ± 5.0
Albumin (g/dL)	4.4 ± 0.3	4.4 ± 0.2	4.6 ± 0.2	4.5 ± 0.2	4.4 ± 0.2	4.6 ± 0.3
Creatinine (mg/dL)	0.9 ± 0.5	0.8 ± 0.2	0.8 ± 0.2	0.8 ± 0.2	0.8 ± 0.2	0.8 ± 0.2
Uric acid (mg/dL)	5.8 ± 1.5	5.3 ± 1.3	5.4 ± 1.4	5.4 ± 1.0	5.5 ± 1.2	5.5 ± 1.4
BUN (mg/dL)	13.4 ± 6.8	13.3 ± 5.2	10.3 ± 3.1	12.5 ± 3.8	12.4 ± 3.4	10.2 ± 3.2
ACR (mg/g cr)^#^	12.8 ± 3.7	10.5 ± 3.2	6.3 ± 2.3	8.0 ± 2.3	6.8 ± 2.6	5.5 ± 2.3
eGFR (mL/min/1.73m^2^)	86.8 ± 20.5	90.1 ± 19.5	104.1 ± 16.7	93.3 ± 17.2	90.7 ± 20.4	105.0 ± 21.3
Triglyceride (mg/dL)^#^	104.6 ± 1.8	94.6 ± 1.8	93.3 ± 1.9	104.6 ± 1.7	102.2 ± 1.7	80.0 ± 1.7
Total cholesterol (mg/dL)	196.4 ± 36.7	202.0 ± 35.1	185.5 ± 30.9	201.8 ± 32.1	195.5 ± 35.6	178.8 ± 29.5
HDL-C (mg/dL)	47.8 ± 13.9	54.2 ± 16.2	49.1 ± 14.2	47.6 ± 12.6	48 ± 13.6	48.6 ± 13.1
LDL-C (mg/dL)	121.1 ± 29.4	121.1 ± 30.4	111.0 ± 26.2	124.9 ± 28.0	119.7 ± 33.7	108.3 ± 25.9
**Disease History**						
Hypertension	36 (45.0)	28 (35.0)	7 (8.8)	24 (30.0)	24 (30.0)	2 (2.5)
Diabetes	12 (15.0)	11 (13.8)	1 (1.3)	11 (13.8)	12 (15.2)	3 (3.8)
Hyperlipidemia	34 (42.5)	31 (38.8)	20 (25.0)	33 (41.8)	33 (41.8)	11 (13.8)
Heart disease	13 (16.3)	12 (15.0)	1 (1.3)	8 (10.0)	8 (10.0)	4 (5.0)
CVA	5 (6.3)	1 (1.3)	0 (0.0)	0 (0.0)	0 (0.0)	0 (0.0)
Cancer	1 (1.3)	8 (10.0)	0 (0.0)	5 (6.3)	4 (5.1)	0 (0.0)

Data were presented as mean±SD for continuous variables or n (%) for categorical variables. BMI: body mass index; WHR: waist to hip ratio; IMT: intima-media thickness; hs-CRP: high-sensitivity C-reactive protein; FPG: fasting plasma glucose; BUN: blood urea nitrogen; *ACR*: albumin to creatinine ratio; eGFR: estimated glomerular filtration rate; CVA: cerebral vascular accident.

#: Geometric mean was presented

### Allele and genotype frequencies in *CRP*, *FGA*, *FGB*, *FGG*, *PON1*, and *EDNRA* genes and their relation with IMT, hs-CRP, and fibrinogen

All 19 SNPs studied in the six genes were tested to be in the HWE (P > 0.05). The allele and genotype distributions for all polymorphisms are shown in Table 2. The associations of *CRP*, *FGA*, *FGB*, *FGG*, *PON1*, and *EDNRA* polymorphisms with IMT, hs-CRP, and fibrinogen were examined. The mean of IMT in individuals carrying the AA variant of SNPs (rs12093699 and rs1130864) of *CRP* gene and SNPs (rs1800789, rs1800790 and rs4220) of *FGB* gene were lower compared with genotype GG. Significant differences in hs-CRP level were found among genotype groups for SNPs rs876537, rs1205 and rs3093059 of *CRP* gene (p< 0.05). Higher hs-CRP concentration was linked with a greater number of G allele of rs876537, rs1205 and rs3093059 polymorphisms. Increasing levels of fibrinogen were also linked with higher numbers of minor alleles of rs1800789, rs1800790, rs1800788, and rs4220 SNPs of *FGB* gene.

**Table 2:** Genotype and allele distributions of study subjects as well as IMT, hs-CRP and fibrinogen distributions according to genotype status^†^.

Chr.	Gene	SNP	Genotype or allele	n (%)	IMT^#^	hs-CRP^#^	Fibrinogen
1	CRP	rs12093699	GG	431 (90.2)	1.20 ± 1.59	0.08 ± 2.85	355.40 ± 67.18
			AG	46 (9.6)	1.10 ± 1.64	0.07 ± 2.65	359.20 ± 74.81
			AA	1 (0.2)	1.04 ± 1.00^a^	0.07 ± 1.00^a^	283.40 ± 0.00^a^
			G	908 (95.0)			
			A*	48 (5.0)			
1	CRP	rs876537	AA	146 (30.6)	1.22 ± 1.58	0.06 ± 2.78	356.10 ± 61.91
			GA	234 (49.1)	1.21 ± 1.65	0.08 ± 2.79^a^	353.50 ± 71.20
			GG	97 (20.3)	1.09 ± 1.50	0.11 ± 2.75^a^	360.00 ± 69.08
			A	526 (55.1)			
			G*	428 (44.9)			
1	CRP	rs1205	AA	145 (30.3)	1.20 ± 1.56	0.06 ± 2.77	356.10 ± 63.82
			GA	237 (49.6)	1.22 ± 1.64	0.08 ± 2.75^a^	354.50 ± 70.09
			GG	96 (20.1)	1.09 ± 1.53	0.10 ± 2.89^a^	357.80 ± 68.93
			A	527 (55.1)			
			G*	429 (44.9)			
1	CRP	rs1130864	GG	418 (88.8)	1.20 ± 1.59	0.08 ± 2.86	355.30 ± 67.54
			AG	51 (10.8)	1.08 ± 1.65	0.07 ± 2.61	356.50 ± 72.56
			AA	2 (0.4)	0.85 ± 1.33^a^	0.13 ± 2.46	330.20 ± 66.28
			G	887 (94.2)			
			A*	55 (5.8)			
1	CRP	rs3093059	AA	322 (67.4)	1.17 ± 1.61	0.07 ± 2.69	356.90 ± 69.17
			GA	141 (29.5)	1.24 ± 1.58	0.11 ± 2.97^a^	357.10 ± 73.02
			GG	15 (3.1)	1.10 ± 1.46	0.11 ± 2.25^a^	337.10 ± 45.20
			A	785 (82.1)			
			G*	171 (17.9)			
4	FGA	rs2070016	AA	295 (61.6)	1.19 ± 1.60	0.08 ± 2.90	348.40 ± 72.01
			GA	155 (32.4)	1.18 ± 1.61	0.08 ± 2.71	368.60 ± 63.74^a^
			GG	29 (6.1)	1.11 ± 1.51	0.09 ± 2.85	372.00 ± 64.79
			A	718 (75.4)			
			G*	234 (24.6)			
4	FGA	rs2070011	AA	132 (27.6)	1.16 ± 1.63	0.08 ± 2.82	351.90 ± 79.41
			GA	230 (48.0)	1.20 ± 1.58	0.08 ± 3.00	352.50 ± 64.77
			GG	117 (24.4)	1.18 ± 1.60	0.08 ± 2.54	368.80 ± 66.03
			A	727 (76.0)			
			G*	229 (24.0)			
4	FGB	rs1800789	GG	274 (57.6)	1.18 ± 1.60	0.08 ± 2.99	348.00 ± 70.99
			AG	170 (35.7)	1.22 ± 1.62	0.08 ± 2.61	366.40 ± 67.87^a^
			AA	32 (6.7)	1.04 ± 1.47^a^	0.08 ± 2.90	376.20 ± 59.39^a^
			G	552 (57.9)			
			A*	402 (42.1)			
4	FGB	rs1800790	GG	279 (58.4)	1.18 ± 1.60	0.08 ± 2.97	348.30 ± 70.76
			AG	169 (35.4)	1.22 ± 1.62	0.08 ± 2.61	365.50 ± 67.89^a^
			AA	30 (6.3)	1.03 ± 1.48^a^	0.08 ± 2.88	381.30 ± 57.88^a^
			G	719 (75.1)			
			A*	239 (24.9)			
4	FGB	rs1800788	AA	163 (34.2)	1.17 ± 1.61	0.07 ± 2.86	345.30 ± 70.83
			GA	226 (47.4)	1.20 ± 1.58	0.08 ± 2.85	357.30 ± 68.37
			GG	88 (18.5)	1.18 ± 1.63	0.08 ± 2.73	370.50 ± 58.34^a^
			A	745 (77.8)			
			G*	213 (22.2)			
4	FGB	rs4220	GG	270 (56.4)	1.19 ± 1.60	0.08 ± 2.98	348.20 ± 71.45
			AG	179 (37.4)	1.21 ± 1.61	0.08 ± 2.59	364.50 ± 66.93^a^
			AA	30 (6.3)	1.03 ± 1.48^a^	0.08 ± 2.97	381.30 ± 57.90^a^
			G	494 (51.6)			
			A*	464 (48.4)			
4	FGG	rs7659024	GG	135 (28.4)	1.19 ± 1.61	0.08 ± 2.60	366.77 ± 64.04
			AG	227 (47.7)	1.20 ± 1.58	0.08 ± 2.97	351.67 ± 65.77
			AA	114 (24.0)	1.16 ± 1.63	0.07 ± 2.84	350.32 ± 75.70
			G	497 (52.2)			
			A*	455 (47.8)			
4	FGG	rs1049636	AA	311 (64.9)	1.20 ± 1.61	0.08 ± 2.86	357.95 ± 70.14
			AG	146 (30.5)	1.13 ± 1.56	0.08 ± 2.74	350.88 ± 69.80
			GG	22 (4.6)	1.40 ± 1.60	0.09 ± 3.11	370.05 ± 60.13
			A	768 (80.2)			
			G*	190 (19.8)			
4	FGG	rs7681423	GG	138 (29.1)	1.19 ± 1.61	0.08 ± 2.63	365.96 ± 63.66
			AG	222 (46.7)	1.20 ± 1.58	0.08 ± 2.97	351.90 ± 66.40
			AA	115 (24.2)	1.16 ± 1.62	0.07 ± 2.82	350.47 ± 75.38
			G	498 (52.4)			
			A*	452 (47.6)			
7	PON 1	rs854555	AA	182 (38.1)	1.19 ± 1.65	0.08 ± 2.72	350.10 ± 63.75
			CA	232 (48.5)	1.18 ± 1.59	0.08 ± 2.94	357.30 ± 72.17
			CC	64 (13.4)	1.22 ± 1.50	0.09 ± 2.83	370.60 ± 75.30
			A	596 (62.3)			
			C*	360 (37.7)			
7	PON 1	rs662	GG	206 (43.3)	1.20 ± 1.63	0.07 ± 2.66	349.30 ± 63.25
			AG	216 (45.4)	1.17 ± 1.58	0.08 ± 3.00	360.00 ± 72.85
			AA	54 (11.3)	1.22 ± 1.51	0.09 ± 2.87	366.60 ± 76.40
			G	628 (66.0)			
			A*	324 (34.0)			
4	EDNRA	rs1395821	AA	184 (38.6)	1.23 ± 1.61	0.09 ± 2.96	356.10 ± 69.93
			GA	229 (48.0)	1.18 ± 1.60	0.08 ± 2.72	354.20 ± 67.88
			GG	64 (13.4)	1.10 ± 1.56	0.07 ± 2.78	359.40 ± 63.14
			A	597 (62.6)			
			G*	357 (37.4)			
4	EDNRA	rs1878406	GG	298 (62.3)	1.16 ± 1.58	0.08 ± 2.81	357.60 ± 67.54
			AG	158 (33.1)	1.22 ± 1.62	0.08 ± 2.84	354.70 ± 70.32
			AA	22 (4.6)	1.29 ± 1.68	0.08 ± 3.00	335.60 ± 51.99
			G	754 (78.9)			
			A*	202 (21.1)			
4	EDNRA	rs5333	AA	300 (62.6)	1.18 ± 1.59	0.08 ± 2.73	354.30 ± 68.38
			GA	161 (33.6)	1.21 ± 1.63	0.08 ± 3.01	361.10 ± 73.76
			GG	18 (3.8)	1.09 ± 1.45	0.11 ± 2.83	348.40 ± 49.75
			A	761 (79.4)			
			G*	197 (20.6)			

Data were presented as n (%) for genotypes and alleles or mean±SD for IMT, hs-CRP, and fibrinogen.

#: Geometric mean was presented.

*: Minor allele.

†: All p-values > 0.05 from Hardy-Weinberg Equilibrium test.

a: p-value < 0.05 for comparing with major-major genotype using GEE in genotypic model.

### Comparison of IMT, serum CRP and fibrinogen level in the recessive models

Linear regression with GEE model was performed for *CRP*, *FGA*, *FGB*, *FGG*, *PON1*, and *EDNRA* gene SNPs separately, under the assumption of recessive models. Regression coefficients (β) and their SE were calculated for minor-minor genotype before and after adjustment for age, sex, BMI, physical activity, smoking, alcohol drinking, and betel nut chewing (Table 3) with minor-major/major-major genotype as the reference genotype. *CRP* gene bearing two minor alleles of SNP rs12093699, rs876537, rs1205, and rs1130864 was associated with IMT in the unadjusted analysis, but their effects were not statistically significant after adjustment for confounding variables. Subjects carrying GG genotype of SNP rs876537 and rs1205 were associated with an increased hs-CRP levels and had an average 1.55- and 1.53-fold higher than subjects carrying GA/GG genotype in the adjusted analysis (S1 Table). In the *FGB* gene, A allele of SNPs rs1800789, rs1800790, and rs4220 were correlated with IMT, and the correlation remained significant after adjustment (Exp. β = 0.89, 0.89, 0.88), but were no significant association in serum FBG concentration and in IMT, hs-CRP, and fibrinogen levels for the SNPs of *FGA*, *FGG*, *PON1*, and *EDNRA* genes with and without adjustment.

**Table 3:** The association between SNPs and IMT, hs-CRP, and fibrinogen in the recessive model^†^.

SNP	Genotype or minor allele	Ln IMT^#^				Ln hs-CRP^#^				Fibrinogen	
		Crude model		Adjusted model^§^		Crude model		Adjusted model^§^		Crude model	Adjusted model^§^
		β (SE)^$^	Exp.(β)^‡^	β (SE)^$^	Exp.(β)^‡^ ^,^	β (SE)^$^	Exp.(β)^‡^	β (SE)^$^	Exp.(β)^‡^ ^,^	β (SE)^$^	β (SE)^$^
CRP											
rs12093699	A	-0.50 (0.01)*	0.61*	0.00 (0.04)	1.00	-0.32 (0.04)*	0.72*	-0.04 (0.12)	0.96	-60.45 (2.35)*	-71.04 (8.91)*
rs876537	G	-0.11 (0.04)*	0.90*	-0.06 (0.04)	0.94	0.37 (0.13)*	1.45*	0.44 (0.12)*	1.55*	4.58 (8.11)	5.26 (7.74)
rs1205	G	-0.10 (0.04)*	0.90*	-0.05 (0.04)	0.95	0.34 (0.13)*	1.41*	0.42 (0.12)*	1.53*	0.42 (8.18)	1.84 (7.76)
rs1130864	A	-0.45 (0.04)*	0.64*	-0.19 (0.12)	0.83	0.38 (0.49)	1.46	0.31 (0.24)	1.36	-19.43 (29.08)	-19.61 (36.7)
rs3093059	G	-0.16 (0.10)	0.85	-0.06 (0.08)	0.95	0.37 (0.21)	1.44	0.37 (0.23)	1.44	-24.71 (11.49)*	-21.89 (12.38)
FGA											
rs2070016	G	-0.07 (0.08)	0.93	-0.03 (0.05)	0.97	0.08 (0.17)	1.09	0.07 (0.14)	1.07	8.52 (11.10)	7.45 (10.47)
rs2070011	G	-0.01 (0.05)	0.99	0.00 (0.04)	1.00	-0.07 (0.10)	0.93	-0.08 (0.09)	0.92	9.96 (6.76)	7.22 (6.65)
FGB											
rs1800789	A	-0.17 (0.07)*	0.84*	-0.12 (0.04)*	0.89*	0.02 (0.17)	1.02	0.04 (0.15)	1.05	12.82 (9.60)	11.53 (9.41)
rs1800790	A	-0.17 (0.07)*	0.84*	-0.12 (0.04)*	0.89*	0.04 (0.17)	1.04	0.05 (0.15)	1.05	18.75 (9.47)	18.25 (9.05)
rs1800788	G	-0.01 (0.05)	0.99	-0.02 (0.04)	0.98	0.02 (0.11)	1.02	0.01 (0.10)	1.01	14.20 (6.72)	12.34 (6.30)
rs4220	A	-0.17 (0.07)*	0.84*	-0.12 (0.04)*	0.88*	-0.01 (0.18)	0.99	0.01 (0.15)	1.01	18.71 (9.49)	19.01 (8.82)
FGG											
rs7659024	A	-0.01 (0.04)	0.99	0.01 (0.04)	1.01	-0.05 (0.11)	0.96	-0.04 (0.10)	0.96	-4.87 (7.86)	-2.59 (7.27)
rs1049636	G	0.14 (0.08)	1.15	0.08 (0.07)	1.09	0.18 (0.29)	1.20	-0.02 (0.25)	0.98	21.92 (12.97)	11.42 (11.60)
rs7681423	A	-0.01 (0.04)	0.99	0.01 (0.04)	1.01	-0.05 (0.11)	0.95	-0.05 (0.10)	0.95	-4.71 (7.82)	-1.80 (7.24)
PON 1											
rs854555	C	0.02 (0.05)	1.02	0.02 (0.05)	1.02	0.15 (0.13)	1.16	0.13 (0.12)	1.14	14.24 (10.61)	14.02 (9.73)
rs662	A	-0.01 (0.06)	0.99	-0.01 (0.04)	0.99	0.18 (0.15)	1.20	0.15 (0.14)	1.16	15.03 (11.90)	14.08 (11.2)
EDNRA											
rs1395821	G	-0.09 (0.05)	0.91	0.02 (0.04)	1.02	-0.24 (0.14)	0.79	-0.17 (0.14)	0.84	-0.80 (9.04)	-0.02 (8.62)
rs1878406	A	0.11 (0.09)	1.11	0.08 (0.07)	1.08	0.00 (0.25)	1.00	0.03 (0.24)	1.03	-13.15 (12.00)	-16.48 (11.75)
rs5333	G	-0.09 (0.09)	0.91	-0.08 (0.05)	0.93	0.43 (0.26)	1.53	0.5 (0.21)	1.65	-1.86 (12.62)	2.62 (11.93)

†: Generalized linear regression with GEE model was performed and using minor-major/major-major genotype as reference genotype.

#: Natural log-transformation for IMT, hs-CRP.

$: β is estimated coefficient of regression model and SE is stand error.

‡: Exponential transformation for estimated coefficient, β.

*: p-value < 0.05.

§: Adjustment of age, gender, obesity, regular exercise, smoking status, alcohol drinking, and betel nut chewing.

The findings from significant interactions of *CRP*, *FGA*, *PON1*, and *EDNRA* gene SNPs with gender and obesity are summarized in Table 4. Significant gene-gender interactions were found for *CRP* gene SNPs rs1130864 and rs3093059 on IMT (P<0.001 and 0.036, respectively), as well as rs1130864 on serum hs-CRP level (P = 0.004) and fibrinogen level (P<0.001) with multivariate adjustment. The interactions of *CRP* gene SNP rs1130864 and rs3093059 with obesity were significant on IMT (P<0.001), serum hs-CRP (P <0.01) and fibrinogen level (P<0.001). In addition, obesity significantly interacted with *FGA* rs2070016, *PON1* rs854555 and *EDNRA* rs5333 on fibrinogen (P = 0.024, 0.036, and <0.001, respectively). Significant gene-regular exercise interactions were not observed.

**Table 4:** Significant gene-gender and obesity interaction on ln IMT, ln hs-CRP, and fibrinogen in recessive model^#^.

Environmental factor	Gene	SNP	P for interaction^a^		
			Ln IMT	Ln hs-CRP	Fibrinogen
Gender	CRP	rs1130864	<0.001	0.004	<0.001
	CRP	rs3093059	0.006		
Obesity	CRP	rs1130864	<0.001	0.004	<0.001
	CRP	rs3093059	<0.001	<0.001	<0.001
	FGA	rs2070016			0.024
	PON1	rs854555			0.036
	EDNRA	rs5333			<0.001

^#^: Generalized linear regression with GEE model considering SNP, age, gender, obesity, regular exercise, smoking status, alcohol drinking, betel nut chewing, and interaction between gender (or obesity) and SNP.

a: FDR p-value.

**Fig 1:**
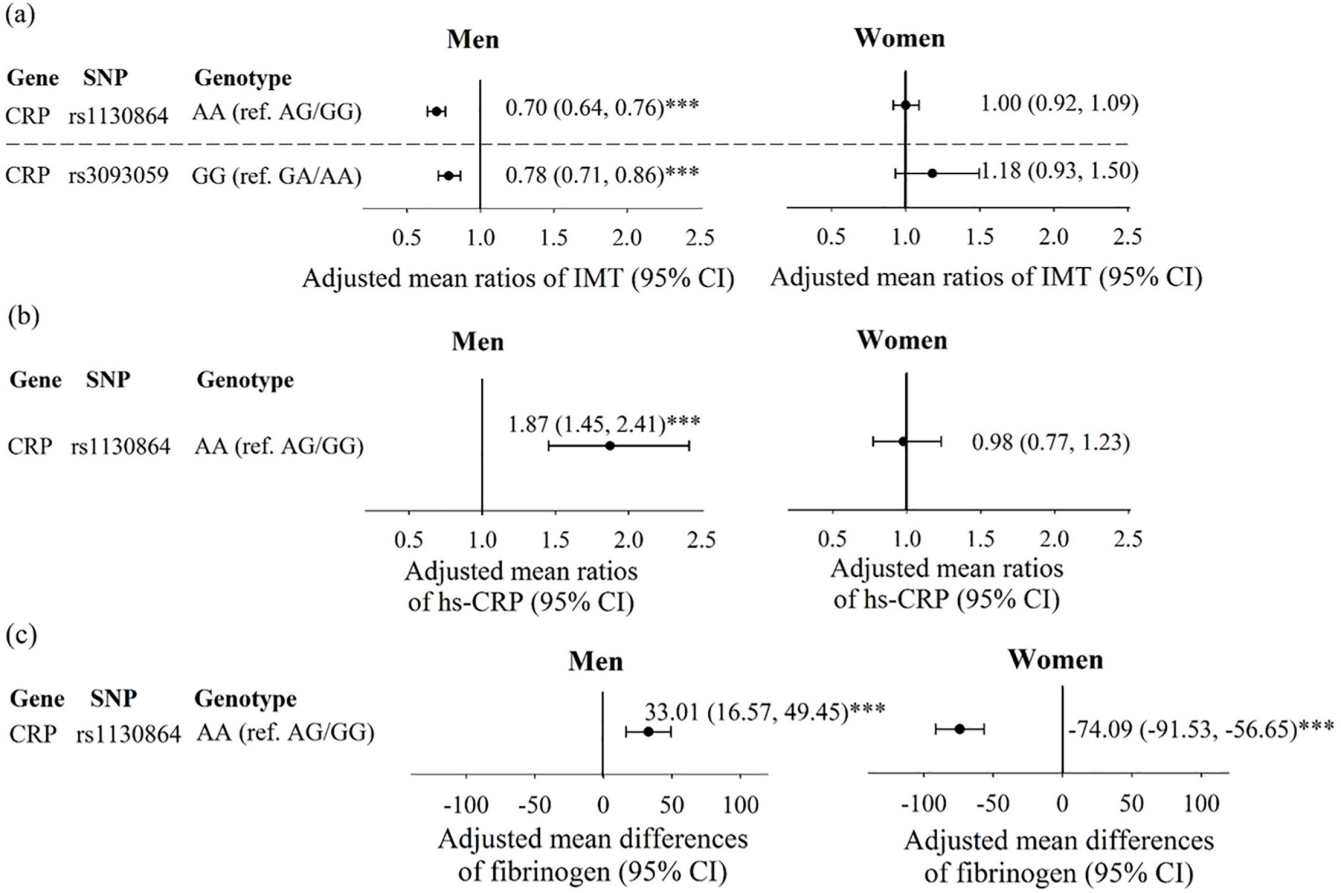
(a) Adjusted mean ratios of IMT, (b) adjusted mean ratios of hs-CRP, and (c) adjusted mean differences of fibrinogen were from generalized linear regression with GEE model considering SNP, age, gender, obesity, regular exercise, smoking status, alcohol drinking, betel nut chewing, and interaction between gender and SNP. *P < 0.05; **P < 0.01; ***P < 0.001.

**Fig 2:**
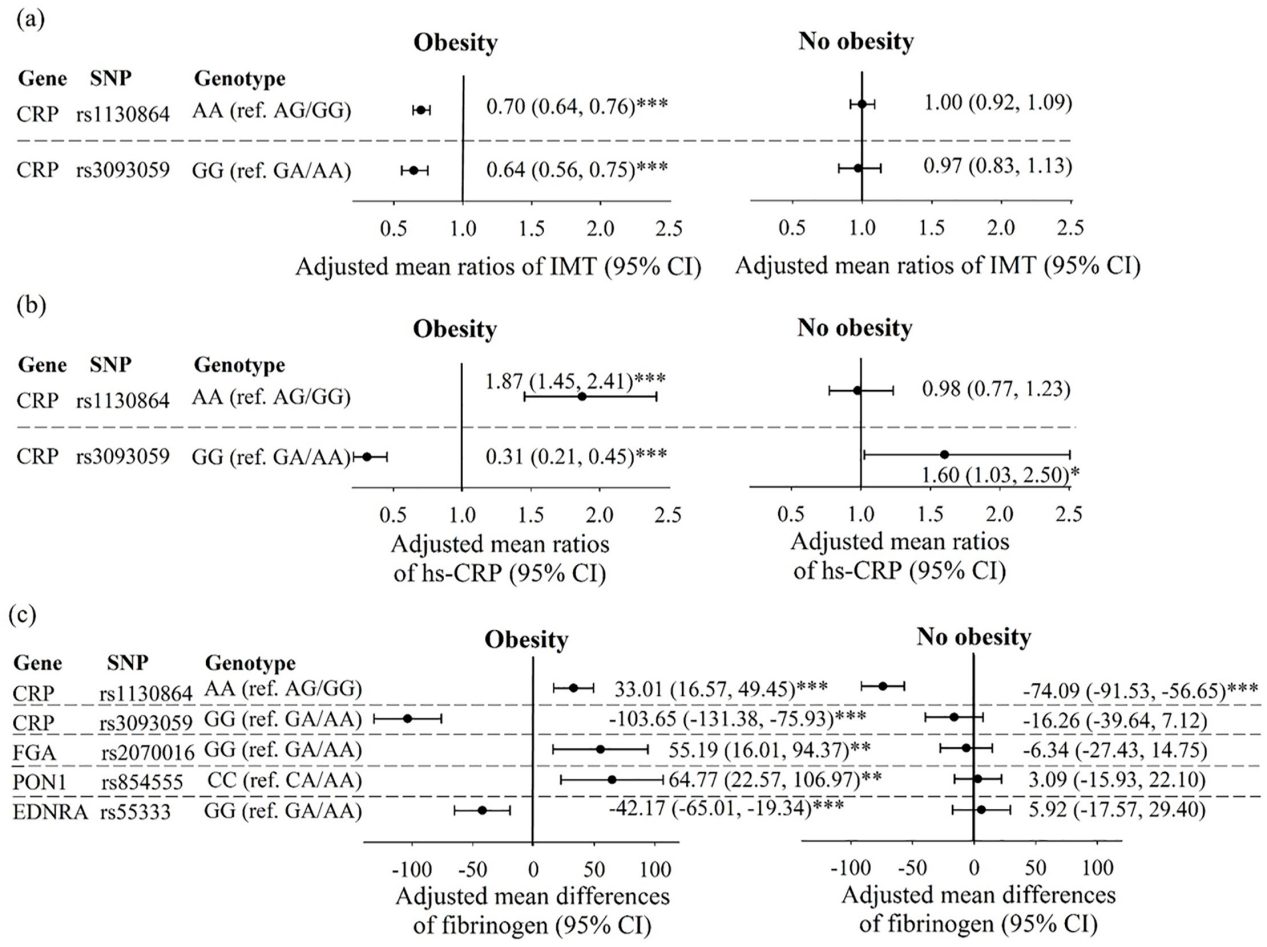
(a) Adjusted mean ratios of IMT, (b) adjusted mean ratios of hs-CRP, and (c) adjusted mean differences of fibrinogen were from generalized linear regression with GEE model considering SNP, age, gender, obesity, regular exercise, smoking status, alcohol drinking, betel nut chewing, and interaction between obesity and SNP. *P < 0.05; **P < 0.01; ***P < 0.001.

To further evaluate the significant interactions identified above, we estimated the adjusted mean differences in fibrinogen and the adjusted mean ratios in carotid IMT and hs-CRP level for *CRP*, *FGA*, *PON1*, and *EDNRA* minor-minor genotype in comparison with minor-major/major-major genotype stratified by gender (Fig 1) and obesity (Fig 2). For gene-gender interaction, the adjusted mean IMT for men carrying minor-minor genotype of *CRP* SNP rs1130864 and rs3093059 were 0.70 and 0.78-fold lower than that for men carrying minor-major/major-major genotype (95% confidence interval (CI): 0.64, 0.76; 0.71, 0.86) (Fig 1A and S1 Table). Men carrying AA genotype of rs1130864 had 1.87-fold higher serum hs-CRP level than men carrying AG/GG genotype (95% CI: 1.45, 2.41) (Fig 1B). On the contrary, we did not observe significant effects for women (Fig 1A and 1B). Among individual with the AA genotype of the *CRP* SNP rs1130864, the adjusted mean difference for fibrinogen levels increased by 33.01 mg/dL (95% CI: 16.57, 49.45) in men and decreased by -74.09 mg/dL (95% CI:-91.53, -56.65) in women, compared with those carrying AG/GG genotype (Fig 1C).

For gene-obesity interaction, obese individuals carrying the minor-minor genotype of *CRP* SNP rs1130864 and rs3093059 had a significantly lower adjusted mean IMT value than those carrying minor-major/major-major genotype (0.70 [0.64, 0.76] and 0.64 [0.56, 0.75], respectively) (Fig 2A and S1 Table). The adjusted mean hs-CRP levels for obese individuals carrying minor-minor genotype of *CRP* SNP rs1130864 and rs3093059 were 1.87-fold higher and 0.31-fold lower than that for obese individuals carrying minor-major/major-major genotype (95% CI:1.45, 2.41; 0.21, 0.45) (Fig 2B). As for fibrinogen levels, the adjusted mean differences in obese individuals carrying the minor-minor genotype of *CRP* rs1130864, *FGA* rs2070016 and *PON* 1 rs854555 was higher by 33.01, 55.19 and 64.77 mg/dL, respectively, compared with those carrying minor-major/major-major genotype (Fig 2C). Whereas individuals carrying the GG genotype of *CRP* SNP rs3093059 and *EDNRA* SNP rs5333 had lower mean values of fibrinogen levels than those carrying the GA/AA genotype among the subjects who obese (Fig 2C). In non-obese individuals, we observed significant effects for *CRP* SNP rs3093059 in hs-CRP levels (Fig 2B) and *CRP* SNP rs1130864 in fibrinogen levels (Fig 2C).

## Discussion

The important role of hs-CRP and fibrinogen concentration and carotid IMT in the occurrence of atherosclerosis and associated complications is well recognized; it is implied that measurements of hs-CRP and fibrinogen levels and carotid IMT may aid in risk stratification and serve as targets for effective prevention of CVD [13, 24]. In this study, we assessed hs-CRP, fibrinogen levels, and 19 SNPs of 6 genes and their correlation with carotid IMT, which is a marker of subclinical cardiovascular disease. Conventional risk factors in this study such as BMI, IMT, smoking, hypertension, heart disease, and CVA were greatest in mean values or prevalence for proband cases than for the other five groups.

Previous studies suggested that inflammation, coagulation, paraoxonase, and endothelial activation pathways may play an important role in carotid atherosclerosis [13–19]. CRP, a major inflammatory biomarker, is associated with higher IMT and ischemic stroke, carotid atherosclerosis, or coronary heart disease [13, 19], although this association has not been confirmed in some studies [25, 44]. *CRP* tag SNP genotype has been associated with increased serum CRP concentration, and a few have been related with decreased hs-CRP levels [45]. Our prior studies indicated that *CRP* gene SNPs rs2794520, rs1205, and rs3093059 were associated with a higher serum CRP level and low handgrip strength in a random sample of elders [46]. This current study analyzed whether five tag SNPs in *CRP* gene were correlated with plasma CRP levels and IMT. Our present results demonstrated the significant relationship between serum CRP concentration and G alleles of SNPs rs876537 and rs1205 with family study design. Higher hs-CRP concentration was linked with a greater number of G alleles of rs876537 and rs1205 polymorphisms. Although SNP rs1130864 was correlated with increased plasma CRP levels in Caucasians [47], we found no correlation between SNPs rs1130864 with CRP levels.

In the recessive model of this study, individuals carrying the GG genotype of *CRP* gene SNP rs876537 and rs1205 were associated with an increased hs-CRP level, and separately had an average 1.55- and 1.53-fold higher levels in the adjusted analyses. Four tag SNP (rs12093699, rs876537, rs1205, and rs1130864) genotypes in *CRP* gene exhibited influence on carotid IMT in the unadjusted analysis, but their effects were not significant in further regression analyses. No evidence of *CRP* variants’ relation to carotid IMT was found in previous studies [27]. Thus, gene-gene and gene-environment interactions may provide useful assistance to the understanding of complexities of genetic determinants of carotid IMT. In this study, significant interaction effects of *CRP* gene SNP rs1130864 and rs3093059 and gender for IMT, hs-CRP and fibrinogen were observed. Men carrying AA genotype of *CRP* rs1130864 and GG genotype of rs3093059 have a 30% and 22% decrease in IMT, (adjusted mean ratios of 0.70 and 0.78, respectively), but carrying AA genotype of *CRP* rs1130864 was associated with a 87% increased serum hs-CRP level. We did not observe significant effects in women. Our findings on the gender difference in the association of IMT and serum hs-CRP level with two SNPs of *CRP* gene suggested that hormones may play an etiologic role on IMT-related cardiovascular disease.

Fibrinogen plays an important role in the pathways of blood coagulation [23]. Many studies have indicated that variation in the fibrinogen structural genes (*FGA*, *FGB* and *FGG*) are related with plasma fibrinogen concentration [23, 25, 26]. Some SNPs of these genetic variants are located in non-coding regions of *FGB*. Most studied fibrinogen polymorphism is SNP rs1800790 (-455G/A) change in the promoter region of *FGB* gene [48–50]. FGB rs1800790 variant elevated plasma fibrinogen levels [26, 51]. Boekholdt et al. suggested that rs1800790 SNP in *FGB* gene was correlated with decreased myocardial infarction risk using the recessive model in a meta-analysis [23]. This SNP exerted protective activity against premature myocardial infarction [52]. Theodoraki et al. proposed that *FGB* rs1800787 and rs1800789 SNPs offered protection for coronary artery disease onset by diminishing about 50% of the risk in homozygotes for the minor alleles [53]. By contrast, Zeng et al. revealed that three SNPs (rs1800790, rs1800787, and rs6050) of the fibrinogen gene were not related with sporadic cerebral hemorrhage when analyzed separately [28]. In our study, minor alleles of rs1800789, rs1800790 and rs4220 SNPs on *FGB* gene were associated with increased plasma fibrinogen levels. However, the effects of these alleles on IMT were the opposite. Homozygotes for the minor alleles of rs1800789, rs1800790 and rs4220 SNPs in *FGB* gene demonstrated significantly lower mean values of IMT after adjustment for confounding factors in the recessive model. These SNPs result in conflicting findings for functionally distinct fibrinogen and IMT, thereby suggesting that the causal pathway for variation in IMT may not be through the alteration of serum fibrinogen levels.

However, the correlation of subclinical atherosclerosis with PON1 activity has been reported [54, 55]. PON1 level and activity are reportedly predictors of cardiovascular diseases, whereas *PON1* genotype was not [56]. Several negative relationships were observed between serum PON-1 activity and carotid IMT in subjects with coronary heart disease [31], hypertension, and ankylosing spondylitis. Previous studies showed inconsistent results regarding the associations between *PON1* SNP rs662 (Q192R) and IMT [21]. A significant difference in mean IMT in female subjects was observed for the *PON1* re662 variants [57]. Another relationship was also observed based on ethnicity for *PON1* rs662 polymorphism in young subjects from Bogalusa Heart Study [57], whereas Karvonen et al. did not observe a significant association between *PON1* rs662 and carotid IMT in the general Caucasian population [58]. Another study also showed a lack of association of *PON1* rs662 with carotid IMT and carotid atherosclerotic plaques [59]. Our study’s findings support the conclusion that *PON1* SNPs were not correlated with carotid IMT.

The EDNRA protein plays a central role in the endothelin-1 pathway and is its function with regard to vasoconstriction [34]. *EDNRA* gene is of great importance in different cardiovascular pathologies [33]. Yasuda et al. shown that *EDNRA* SNP rs5333 was positively associated with mean IMT, and this association was only apparent in male hypertensive patients [60]. Li et al. indicated the interaction of smoking with *EDNRA* rs6841473 on left carotid IMT in Africa Americans [15]. Lopez-Mejias et al. did not confirm the association between the *EDNRA* rs1878406 polymorphisms and carotid IMT in rheumatoid arthritis with subclinical atherosclerosis and cardiovascular problem [16]. Our previous study showed that *EDNRA* gene rs1395821 significantly interacted with regular exercise and rs5333 with obesity on IMT [35]. This study’s results identified a significant interaction of obesity with SNP rs5333 in the *EDNRA* gene on fibrinogen. Our findings provided the knowledge of how life-style associated risk factors may interact with EDNRA gene variants in IMT risk, which would be useful for research on carotid IMT risk prediction and prevention in the general population.

## Conclusion

Our research was a community-based family study with detailed systematic examination of our quantitative phenotype. One limitation that must be explained is the relatively small sample size. The number individuals who underwent carotid IMT measurements (480 individuals) may be too small to provide sufficient statistical power for a stratified analysis. Our results provide evidence for the main effects of the *FGB* genes on IMT, for the interaction of *CRP* SNP rs1130864 and rs3093059 with gender on IMT, and with obesity on IMT, hs-CRP and fibrinogen levels. These results support the hypothesis that inflammatory genes are involved in atherosclerosis, most likely via complex gene-gender and gene-obesity interactions.

## Supporting information

S1 Data (DOCX)

S1 Table A summary table of describing significant regression analysis and interaction results simply. (DOCX)
