# Supplementary material for: Evaluation of single nucleotide polymorphisms in 6 candidate genes and carotid intima-media thickness in community-dwelling residents
Source: PLoS One. 2020 Mar 26;15(3):e0230715. doi: 10.1371/journal.pone.0230715 (PMC7098559; doi:10.1371/journal.pone.0230715)
Supplement: S1 Data — (DOCX) [file pone.0230715.s001.docx]

The questionnaire used in this study was shown below, including English and Chinese versions.

I. English version

| **Basic information**  Gender: 🞎1. Male 🞎0. Female  Birth dates: Year Month Day  **Behaviors**  Do you have a habit of cigarette smoking?  (Ever or current cigarette smoking: at least half a pack of cigarettes per week (10 cigarettes) for at least 6 months)  🞎0. No 🞎1. Yes  🞎2. Ever, quit cigarette smoking (more than one year)  Do you have a habit of alcohol drinking?  (Ever or current alcohol drinking: at least once a week for at least 6 months)  🞎0. No 🞎1. Yes  🞎2. Ever, quit alcohol drinking (more than one year)  Do you have a habit of betel nut chewing?  (Ever or current betel nut chewing: betel nut chewing for at least 6 months)  🞎0. No 🞎1. Yes  🞎2. Ever, quit betel nut chewing (more than one year)  Do you have a habit of regular exercise? (Ever or current regular exercise: participated in regular leisure-time activities for at least 30 min per week for at least 6 months, including taking a walk, fast walking, etc.)  🞎0. No 🞎1. Yes  **Personal history of diseases:** please check if you have been diagnosed by a doctor or informed of the following diseases.   \| Disease \| Have you ever been diagnosed with the disease? \| Are you taking medication? \| \| --- \| --- \| --- \| \| Heart disease  (1) Myocardial infarction  (2) Angina pectoris  (3) Have received coronary artery bypass graft  (4) Have received percutaneous coronary stent intervention  (5) Have received percutaneous transluminal coronary angioplasty  (6) Heart failure  (7) Atrial fibrillation \| 🞎1. Yes 🞎0. No  🞎1. Yes 🞎0. No  🞎1. Yes 🞎0. No  🞎1. Yes 🞎0. No  🞎1. Yes 🞎0. No  🞎1. Yes 🞎0. No  🞎1. Yes 🞎0. No \| 🞎1. Yes 🞎0. No  🞎1. Yes 🞎0. No  🞎1. Yes 🞎0. No  🞎1. Yes 🞎0. No  🞎1. Yes 🞎0. No  🞎1. Yes 🞎0. No  🞎1. Yes 🞎0. No \| \| Stroke \| 🞎1. Yes 🞎0. No \| 🞎1. Yes 🞎0. No \| \| Hypertension \| 🞎1. Yes 🞎0. No \| 🞎1. Yes 🞎0. No \| \| [Hyperlipidemia](https://www.google.com/search?q=Hyperlipidemia&spell=1&sa=X&ved=2ahUKEwjXk4ySqZbmAhWtxIsBHXDyDoUQBSgAegQICxAr)  (1) Abnormal triglyceride  (2) Abnormal total cholesterol  (3) Abnormal HDL-C \| 🞎1. Yes 🞎0. No  🞎1. Yes 🞎0. No  🞎1. Yes 🞎0. No \| 🞎1. Yes 🞎0. No  🞎1. Yes 🞎0. No  🞎1. Yes 🞎0. No \| \| Diabetes Mellitus  (1) Abnormal/higher fasting plasma glucose or postprandial glucose \| 🞎1. Yes 🞎0. No \| 🞎1. Yes 🞎0. No \| \| Cancer \| 🞎1. Yes 🞎0. No \| 🞎1. Yes 🞎0. No \| |
| --- | --- | --- | --- | --- | --- | --- | --- | --- | --- | --- | --- | --- | --- | --- | --- | --- | --- | --- | --- | --- | --- |

II. Chinese version

| **基本資料**  性別： 🞎1.男 🞎0.女  出生日期： 年 月 日  **健康行為**  請問您有無抽菸的習慣？  (曾經或現在有抽菸習慣：指每週至少抽半包菸(10支)，且超過六個月以上)  🞎0.無 🞎1.有  🞎2.以前有，但已戒掉（戒除抽菸習慣至少超過一年以上，才算是戒菸）  請問您有無飲酒的習慣？  （曾經或現在有飲酒習慣：指每週至少飲酒一次，且連續六個月以上）  🞎0.無 🞎1.有  🞎2.以前有，但已戒掉(戒除飲酒習慣超過一年以上，才算是戒酒)  請問您有無嚼食檳榔的習慣？  (曾經或現在有嚼食檳榔習慣：指嚼檳榔至少六個月以上)  🞎0.無 🞎1.有  🞎2.以前有，但已戒掉(戒除嚼檳榔習慣超過一年以上，才算是戒掉)  請問您有無規律運動的習慣？（曾經或現在有運動的習慣：每週至少運動一次， 每次至少30分鐘，而且連續六個月以上，運動項目可包括散步、快走等）  🞎0.無 🞎1.有  **一般病史**：請問您本人是否曾被醫師診斷出或被醫師告知患有下列疾病，請打勾。   \| 疾病名稱 \| 是否被診斷出有此疾病 \| 目前是否服用藥物 \| \| --- \| --- \| --- \| \| 心臟病  (1)心肌梗塞  (2)心絞痛（狹心症）  (3)曾接受冠狀動脈繞道手術  (4)曾接受冠狀動脈支架手術  (5)曾接受冠狀動脈汽球擴張術  (6)心臟衰竭  (7)心房顫動 \| 🞎1.是 🞎0.否  🞎1.是 🞎0.否  🞎1.是 🞎0.否  🞎1.是 🞎0.否  🞎1.是 🞎0.否  🞎1.是 🞎0.否  🞎1.是 🞎0.否 \| 🞎1.是 🞎0.否  🞎1.是 🞎0.否  🞎1.是 🞎0.否  🞎1.是 🞎0.否  🞎1.是 🞎0.否  🞎1.是 🞎0.否  🞎1.是 🞎0.否 \| \| 腦中風 \| 🞎1.是 🞎0.否 \| 🞎1.是 🞎0.否 \| \| 高血壓 \| 🞎1.是 🞎0.否 \| 🞎1.是 🞎0.否 \| \| 高血脂症  (1)三酸甘油脂(中性脂肪)過高？  (2)總膽固醇過高？  (3)高密度膽固醇(好膽固醇)過低？ \| 🞎1.是 🞎0.否  🞎1.是 🞎0.否  🞎1.是 🞎0.否 \| 🞎1.是 🞎0.否  🞎1.是 🞎0.否  🞎1.是 🞎0.否 \| \| 糖尿病  (1)空腹或飯後血糖異常或過高？ \| 🞎1.是 🞎0.否 \| 🞎1.是 🞎0.否 \| \| 癌症 \| 🞎1.是 🞎0.否 \| 🞎1.是 🞎0.否 \| |
| --- | --- | --- | --- | --- | --- | --- | --- | --- | --- | --- | --- | --- | --- | --- | --- | --- | --- | --- | --- | --- | --- |
