# Supplementary material for: Evaluation of single nucleotide polymorphisms in 6 candidate genes and carotid intima-media thickness in community-dwelling residents
Source: PLoS One. 2020 Mar 26;15(3):e0230715. doi: 10.1371/journal.pone.0230715 (PMC7098559; doi:10.1371/journal.pone.0230715)
Supplement: S1 Table — (DOCX) [file pone.0230715.s002.docx]

Supplemental Table 1. A summary table of describing significant regression analysis and interaction results simply

| Gene | SNP | Genotype | IMT | hs-CRP | Fibrinogen |
| --- | --- | --- | --- | --- | --- |
| *CRP* | rs876537 | GG (ref. GA/AA) | － | ^‡^Subjects carrying GG genotype had 1.55-fold higher on hs-CRP than those carrying GA/AA genotype. | － |
| *CRP* | rs1205 | GG (ref. GA/AA) | － | ^‡^Subjects carrying GG genotype had 1.53-fold higher on hs-CRP than those carrying GA/AA genotype. | － |
| *CRP* | rs1130864 | AA (ref. AG/GG) | ^#^Gene-gender interaction: men with AA genotype had 0.70-fold lower on IMT than men with AG/GG genotype.  ^§^Gene-obesity interaction: obese individuals with AA genotype had 0.70-fold lower on IMT than those with AG/GG genotype. | ^#^Gene-gender interaction: men with AA genotype had 1.87-fold higher on hs-CRP than men with AG/GG genotype. ^§^Gene-obesity interaction: obese individuals with AA genotype had 1.87-fold higher on hs-CRP than those with AG/GG genotype. | ^#^Gene-gender interaction: (1) men with AA genotype had higher fibrinogen (increased by 33.01 mg/dL) than men with AG/GG genotype; (2) women with AA genotype had lower fibrinogen (decreased by -74.09 mg/dL) than women with AG/GG genotype. ^§^Gene-obesity interaction: (1) obese individuals with AA genotype had higher fibrinogen (increased by 33.01 mg/dL) than those with AG/GG genotype; (2) non-obese individuals with AA genotype had lower fibrinogen (decreased by -74.09 mg/dL) than those with AG/GG genotype. |
| *CRP* | rs3093059 | GG (ref. GA/AA) | ^#^Gene-gender interaction: men with GG genotype had 0.78-fold lower on IMT than men with GA/AA genotype. ^§^Gene-obesity interaction: obese individuals with GG genotype had 0.64-fold lower on IMT than those with GA/AA genotype. | ^§^Gene-obesity interaction: (1) obese individuals with GG genotype had 0.31-fold lower on hs-CRP than those with GA/AA genotype; (2) non-obese individuals with GG genotype had 1.60-fold higher on hs-CRP than those with GA/AA genotype. | ^§^Gene-obesity interaction: obese individuals with GG genotype had lower fibrinogen (decreased by -103.65 mg/dL) than those with GA/AA genotype. |
| *FGB* | rs1800789 | AA (ref. AG/GG) | ^‡^Subjects carrying AA genotype had 0.89-fold lower on IMT than those carrying AG/GG genotype. | － | － |
| *FGB* | rs1800790 | AA (ref. AG/GG) | ^‡^Subjects carrying AA genotype had 0.89-fold lower on IMT than those carrying AG/GG genotype. | － | － |
| *FGB* | rs4220 | AA (ref. AG/GG) | ^‡^Subjects carrying AA genotype had 0.88-fold lower on IMT than those carrying AG/GG genotype. | － | － |
| *FGA* | rs2070016 | GG (ref. GA/AA) | － | － | ^§^Gene-obesity interaction: obese individuals with GG genotype had higher fibrinogen (increased by 55.19 mg/dL) than those with GA/AA genotype. |
| *PON1* | rs854555 | CC (ref. CA/AA) | － | － | ^§^Gene-obesity interaction: obese individuals with CC genotype had higher fibrinogen (increased by 64.77 mg/dL) than those with CA/AA genotype. |
| *EDNRA* | rs5333 | GG (ref. GA/AA) | － | － | ^§^Gene-obesity interaction: obese individuals with GG genotype had lower fibrinogen (decreased by -42.17 mg/dL) than those with GA/AA genotype. |

We used generalized linear regression with GEE models to perform our regression analyses.

^‡^: Multiple regression analysis with adjustment of age, gender, obesity, regular exercise, smoking status, alcohol drinking, and betel nut chewing.

^#^: Adjusted mean ratio of IMT (or hs-CRP; or adjusted mean difference of fibrinogen) from model considering SNP, age, gender, obesity, regular exercise, smoking status, alcohol drinking, betel nut chewing, and interaction between gender and SNP.

^§^: Adjusted mean ratio of IMT (or hs-CRP; or adjusted mean difference of fibrinogen) from model considering SNP, age, gender, obesity, regular exercise, smoking status, alcohol drinking, betel nut chewing, and interaction between obesity and SNP.
